# *Syzygium pyneei* (Myrtaceae), a new critically endangered endemic species from Mauritius

**DOI:** 10.3897/phytokeys.46.9039

**Published:** 2015-02-05

**Authors:** James W. Byng, F. B. Vincent Florens, Cláudia Baider

**Affiliations:** 1 School of Biological Sciences, University of Aberdeen, Aberdeen, AB24 3UU, UK; 2 Herbarium, Royal Botanic Gardens, Kew, Richmond, Surrey TW9 3AB, UK; 3 Department of Biosciences, University of Mauritius, Réduit, Mauritius; 4 The Mauritius Herbarium, Agricultural Services, Ministry of Agro-Industry and Food Security; R. E. Vaughan Building, Réduit, Mauritius

**Keywords:** *Syzygium*, endemic, Mascarenes, Myrtaceae

## Abstract

A new species of *Syzygium* Gaertn. (Myrtaceae), *Syzygium pyneei* Byng, V. Florens & Baider, is described from Mondrain Reserve on the island of Mauritius. This species is endemic to the island and differs from any other species by its combination of cauliflory, relatively large flowers, light green to cream hypanthium, light pink stamens, short thick petioles, coriaceous leaves and round, cuneate or sub-cordate to cordate leaf bases. *Syzygium pyneei* Byng, V. Florens & Baider is known from only two individuals from the type locality and merits the conservation status of Critically Endangered (CR C2a(i,ii); D).

## Introduction

*Syzygium* Gaertn. is the largest genus in Myrtaceae with about 1200 species distributed in the Old World tropics and subtropics (WCSP 2014). Scott (1990) described fourteen species native to Mauritius after which Bosser and Florens (2000) described another new *Syzygium* species, *Syzygium guehoi* Bosser & Florens, from the island. During recent morphological and molecular work on the genus in the African-Indian Ocean region by the first author it was noted that samples from Mondrain Reserve were quite different from any other known taxa. Therefore, the species is described here as a new species.

## Taxonomic treatment

### Syzygium pyneei

Taxon classificationPlantaeMyrtalesMyrtaceae

Byng, V. Florens & Baider sp. nov.

urn:lsid:ipni.org:names:77145080-1

Figures 1 , 2

#### Type.

Mauritius, Mondrain Reserve, 30–35 m from main gate on left of path at 20°19.597'S; 57°27.241'E, 24 Nov 2006, G. D’Argent & K. Pynee MAU 25014 (holotype: MAU! [MAU 0014027; spirit MAU 0014029 fl.buds, fl.]).

#### Diagnosis.

A cauliflorous species with relatively large flowers (> 2 cm long), light green to cream hypanthium, light pink stamens, short, thick petioles (4–8 mm long), and round, cuneate or sub-cordate to cordate leaf bases. The species could be confused with *Syzygium mauritianum* but differs in the usually longer petioles, the variable leaf base and light green to cream hypanthium. *Syzygium pyneei* could also be confused with *Syzygium cymosum* but differs from the latter species in the light green hypanthium, sepals 4–5 mm long and coriaceous leaves.

#### Description.

Glabrous shrub to 3.5 m; bark grey to sometimes creamy-pink; branchlets terete, grey to reddish-brown. *Leaves* drying pale green above, light brown below; coriaceous, 10‒15 × 4.5‒9 cm, elliptic, oval-elliptic or oblong-elliptic, base round, cuneate or sub-cordate to cordate, apex acute to shortly acuminate, margin flat; 10‒18 secondary vein pairs, 3–16 mm apart, prominent on both sides, tertiary veins few, faint; inner intramarginal vein 2–4 mm from leaf margin, outer intramarginal faint, 1–2 mm from leaf margin; petiole 4‒8 mm long, robust, green when young, reddish-brown when old, 2–3 mm diameter. *Inflorescences* cauliflorous, ca. 6 cm long, axes terete, flowers up to 6, in clusters of 1–3; bracts and bracteoles deltoid, ca. 2 mm long, caducous. *Flowers* ca. 2 cm long; pseudostalk ca. 2 mm long; hypanthium 12–13 × 7–11 mm, pyriform, light green to cream; sepals 4–5 × 2–3 mm, obtusely triangular or obtuse; petals 8–9 × ca. 6 mm, orbicular; stamens 12‒15 mm long, light pink; anthers ca. 1 mm; ovules few per locule; style 7‒18 mm long. *Fruits* pyriform, 20 mm long × larger diameter 15.8‒19.3 mm and smaller diameter (near calyx disc) 12.5‒14 mm; colour not recorded, surface with few hairs. *Seeds* 1‒2, globular when 1, half-moon shape when 2; 11.2‒11.6 mm × 10.7‒12.3 × 11‒11.5 mm when globular or same height but 10 × 6 mm diameter when half-moon shape, testa bullate; not seen fresh.

**Figure 1.**
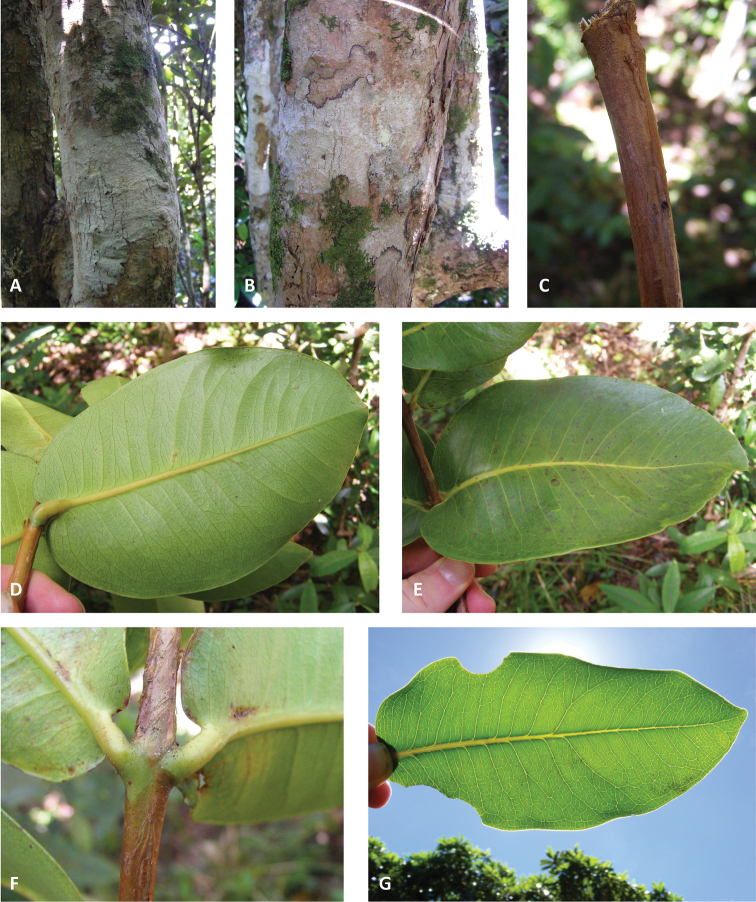
Vegetative characters. **A** and **B** bark **C** close-up of branchlet **D** lower leaf surface **E** upper leaf surface **F** petioles **G** leaf venation. (**A** and **G** Byng 83; **B–F** Byng 84).

#### Flowering and fruiting.

The species was reportedly flowering for the first time in about 20 years (G. D’Argent pers. obs.) in November 2006. By 1 December 2006 most flowers (80%) were found on the ground and a few fruits were collected on 19 January 2007 after several visits monitoring the population.

**Figure 2.**
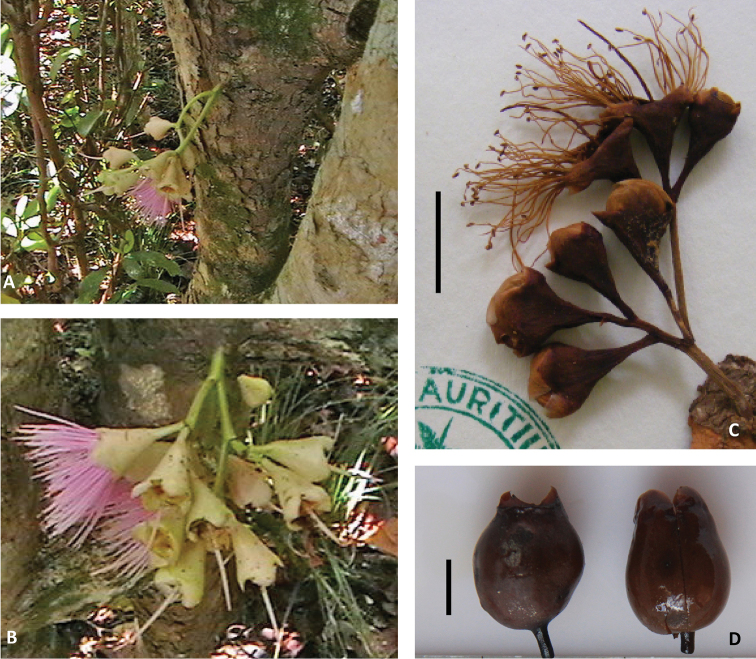
Floral and fruit characters. **A** and **B** Sole recorded images of flowering event **C** Close-up of dried inflorescence **D** Close-up of two fruits. (**A–C** D’Argent & Pynee MAU 25014; **D** D’Argent & K. Pynee MAU 26448; **A** and **B** courtesy of Kersley Pynee). Scale bar = 1 cm.

#### Distribution.

This species is only known from Mauritius, and has not been recorded outside Mondrain Reserve. Only two individuals have been recorded and no seedlings have been seen.

#### Ecology.

This species grows in a ridge forest, not fully exposed to sun and wind, at an elevation of around 520 m.

#### Etymology.

*Syzygium pyneei* is named after Kersley Pynee who co-collected the type specimen and is a prominent local botanist.

#### Additional specimens examined.

MAURITIUS, Mondrain Reserve, 10 Nov 2006, G. D’Argent & K. Pynee MAU 25013 (MAU [0014026]); 01 Dec 2006, G. D’Argent & K. Pynee MAU 26447, fl., (spirit, MAU [0014026]); 19 Jan 2007, G. D’Argent & K. Pynee MAU 26448, fr., (spirit, MAU [0014030]); 31 Mar 2011, J.W. Byng 83 (K, MAU) & 84 (E, MAU).

#### Discussion.

*Syzygium pyneei* most closely resembles *Syzygium cymosum* (Lam.) DC., and *Syzygium mauritianum* J. Guého & A.J. Scott, sharing the cauliflorous habit, pinkish flowers and large leaves (≥ 10 cm long). *Syzygium mauritianum* individuals have sessile to very short petioles (0–5 mm long), bright pinkish-red hypanthia, usually very large leaves ((10–)17–30 cm long) and strongly cordate leaf bases, in contrast to the 5–8 mm long petioles, light green to cream hypanthium and round, cuneate or sub-cordate to cordate leaf bases of *Syzygium pyneei*. Scott (1990) suggested *Syzygium cymosum* was probably extinct on Mauritius, as no specimens had been collected for many years, but extant on La Réunion. Specimens of *Syzygium pyneei* were originally thought to be related to *Syzygium cymosum* when the flowering individual was first seen but *Syzygium pyneei* differs by the light green to cream hypanthium (vs. light pink), sepals 4–5 mm long (vs. ca. 1 mm) and coriaceous leaves (vs. chartaceous).

In addition, molecular data (Byng unpublished data) suggests *Syzygium pyneei* is most closely related to *Syzygium guehoi*; morphologically both species have several-flowered, cauliflorous inflorescences and are distributed on the western part of the island. However, *Syzygium pyneei* has larger leaves, flowers and fruits and *Syzygium guehoi* are much larger individuals, growing up to 15 m tall with conspicuous red petioles.

#### Conservation status.

This species is currently known from only two individuals. The population at Mondrain is protected within a private reserve of around 5 ha, which has been cleared of invasive alien plants and fenced against alien deer. Outside the fenced area, which is potential habitat for further individuals, the forest is dominated by alien plants, notably *Psidium cattleianum* Afzel. ex Sabine, the main invader in moist to wet forests of the island and a species known to be very detrimental to native plants on Mauritius (Baider and Florens 2011; Monty et al. 2013). The reserve is adjacent to deer grazing lands to the east. *Syzygium pyneei* should be considered Critically Endangered (CR C2a(i,ii); D) according to the IUCN Red List Criteria (IUCN 2001).

## Supplementary Material

XML Treatment for Syzygium pyneei
